# Cardiovascular risk factors predate the onset of symptoms of rheumatoid arthritis: a nested case–control study

**DOI:** 10.1186/s13075-017-1351-8

**Published:** 2017-06-30

**Authors:** Heidi Kokkonen, Hans Stenlund, Solbritt Rantapää-Dahlqvist

**Affiliations:** 1 Department of Public Health and Clinical Medicine, Rheumatology, Umeå University, Building 6M, 901 87 Umeå, Sweden; 2 Department of Public Health and Clinical Medicine, Epidemiology and Global Health, Umeå University, Umeå, Sweden

**Keywords:** Rheumatoid arthritis, Cardiovascular disease, Body mass index, Apolipoproteins, Diabetes mellitus, Smoking

## Abstract

**Background:**

Patients with rheumatoid arthritis (RA) are at increased risk of developing cardiovascular disease (CVD). Our aim was to evaluate the impact of factors related to CVD, such as smoking, lipid levels, hypertension, body mass index (BMI) and diabetes, in individuals prior to the onset of symptoms of RA.

**Methods:**

A nested case–control study was performed including data from 547 pre-symptomatic individuals (i.e. individuals who had participated in population surveys in northern Sweden prior to onset of symptoms of RA, median time to symptom onset 5.0 (interquartile range 2.0–9.0) years) and 1641 matched controls. Within the survey, health examinations prior to symptom onset were performed, blood samples were analysed for plasma glucose and lipids, and data on lifestyle factors had been collected with a questionnaire. CVD risk factors were extracted and further analysed with conditional logistic regression models for association with subsequent RA development, including hypertension, apolipoprotein (Apo)B/ApoA1 ratio, BMI, diabetes and smoking habits.

**Results:**

Smoking and BMI ≥ 25 (odds ratio (OR) (95% confidence interval (CI)) =1.86 (1.48–2.35) and OR = 1.28 (1.01–1.62), respectively) were associated with increased risk for future RA development. In women, elevated ApoB/ApoA1 ratio (OR = 1.36 (1.03–1.80)) and smoking (OR = 1.82 (1.37–2.41)) were significantly associated with being pre-symptomatic for RA, whilst in men smoking (OR = 1.92 (1.26–2.92)) and diabetes (OR = 3.62 (95% CI 1.13–11.64)) were significant. In older (>50.19 years) individuals, only smoking (OR = 1.74 (1.24–2.45)) was significantly associated with increased risk of future RA, whereas in younger individuals the significant factors were elevated ApoB/ApoA1 ratio (OR = 1.39 (1.00–1.93)), BMI ≥ 25.0 (OR = 1.45 (1.04–2.02)) and smoking (OR = 2.11 (1.51–2.95)). Pre-symptomatic individuals had a higher frequency of risk factors: 41.5% had ≥3 compared with 30.4% among matched controls (OR = 2.81 (1.78–4.44)).

**Conclusions:**

Several risk factors for CVD were present in pre-symptomatic individuals and significantly associated with increased risk for future RA. These factors differed in women and men. The CVD risk factors had a greater impact in younger individuals. These results urge an early analysis of cardiovascular risk factors for proposed prevention in patients with early RA.

## Background

Patients with rheumatoid arthritis (RA) have an increased risk of developing several co-morbidities compared with the general population, the commonest co-morbidity being cardiovascular disease (CVD) [1]. Patients with RA also have an increased risk of mortality due to CVD [2–5]. The prevalence of CVD is comparable with that in patients with type 2 diabetes [6]. The aetiology of this increased morbidity and mortality is not fully understood, although traditional risk factors (i.e. cigarette smoking, hypertension, diabetes mellitus and elevated levels of low-density lipoproteins) as well as the inflammatory burden are involved [1, 2, 7, 8].

In a retrospective cohort study, RA patients had a significantly higher risk of hospitalization for either acute or unrecognized myocardial infarction (MI) prior to developing RA [9]. However, in another study no increased risk of ischaemic heart disease, MI or angina pectoris before the onset of RA was reported [10]. The risk of MI was increased, being apparent 1–4 years following diagnosis of RA [11]. In a nested case–control study analysing the presence of risk factors for CVD in individuals prior to development of inflammatory polyarthritis (IP), the only risk factor associated with IP development was smoking [12]. Other CVD risk factors (e.g. total cholesterol, low-density lipoprotein (LDL) cholesterol, systolic and diastolic blood pressure and obesity) were not increased prior to onset of IP [12]. Conversely, a study of individuals prior to onset of symptoms of RA reported a more atherogenic lipid profile with higher levels of total cholesterol, triglycerides and apolipoprotein (Apo)B in addition to lower levels of high-density lipoprotein (HDL) cholesterol compared with control subjects, independent of RF and ACPA, and marginally affected by CRP [13]. A contradictory study reported a significant decrease in levels of total cholesterol as well as LDL-cholesterol and HDL-cholesterol during the 5 years prior to diagnosis of RA compared with non-RA controls [14].

Obesity has been identified as a risk factor for RA [15] and recently we identified that abdominal obesity was associated with an increased risk of subsequent development of RA [16]. After stratification for sex, this association was restricted to men with an early disease onset [16].

The presence of insulin resistance after onset of RA is known, whilst the presence of diabetes before RA onset has been sparsely reported. In one study, the presence of diabetes was associated with increased risk for RA development in women [17].

The aim of this study was to compare lifestyle factors, lipid levels, presence of hypertension and diabetes in individuals prior to onset of symptoms of RA with matched controls in a nested case–control design based on a population-based intervention project from northern Sweden.

## Methods

### Study cohorts

This study was based on information from the Västerbotten Intervention Programme (VIP) and the Northern Sweden Multinational Monitoring of Trends and Determinants in Cardiovascular Disease (MONICA). The VIP, details of which have been described previously [18], is a population-based study aimed at reducing morbidity and mortality due to CVD and diabetes in northern Sweden. Briefly, since 1991, all individuals aged 40, 50 and 60 years living in the county of Västerbotten are invited to participate in a health assessment at their local primary care centre; the participation rate throughout has been approximately 60% [19]. All participants complete a health questionnaire regarding socioeconomic and demographic status, educational level (analysed as no academic education/university vs academic education/university), self-reported health (including medication) and lifestyle (e.g. exercise and smoking habits). Blood samples were drawn and analysed for total cholesterol, triglycerides, HDL-cholesterol, and LDL-cholesterol according to routine protocols, with excess samples stored at –80° for future analysis. Plasma glucose (baseline) was measured in an overnight fasting sample together with a sample 2 hours after the intake of 75 g anhydrous glucose. Blood pressure was measured twice, the mean value reported. Waist circumference (cm) was measured and the body mass index (BMI; kg/m^2^) was calculated.

Details for the registration and survey procedures in the Northern Sweden MONICA project including the two most northern counties have been described previously [20]. Briefly, since 1986, seven population surveys including a physical examination and a questionnaire similar to that used in the VIP have been performed. At examination, blood samples were drawn for analysis, as in the VIP project.

### Identification of individuals before onset of symptoms of RA

In 2014, the registers of patients attending the Department of Rheumatology, University Hospital, Umeå, Sweden (the only department within the county) and fulfilling the 1987 ARA criteria for diagnosis of RA since 1995 were co-analysed with those from the VIP and MONICA. This linkage identified 557 individuals having participated in these cohorts prior to the onset of symptoms of joint disease (“pre-symptomatic individual” or case). For each case, three controls were selected randomly, matched for sex, year of birth, participation in the VIP or MONICA and living in a rural or urban area, resulting in 1671 controls without known RA until the end of 2014. In a second evaluation of the cases, 10 of the pre-symptomatic individuals and corresponding controls were excluded due to having experienced symptoms prior to participation in the VIP, resulting in 547 pre-symptomatic individuals (498 (91%) from the VIP and 49 (9%) from MONICA) and 1641 controls (1494 (91%) from the VIP and 147 (9%) from MONICA) identified for inclusion in this study. The median (Q1–Q3) age of the pre-symptomatic individuals was 50.19 (45.50–59.95) years and for controls was 50.26 (45.94–59.97) years, and the median pre-dating time to symptom onset was 5.0 (2.0–9.0) years. The median (Q1–Q3) duration of follow-up for the controls was 17.0 (14.0–21.0) years.

### Analyses of ApoA1 and ApoB

ApoA1 (g/L) and ApoB (g/L) were analysed from stored samples using an immunoturbimetric method (Cobas 8000 instrument; Roche Diagnostics Scandinavia AB).

### Statistical analyses

Statistical calculations were performed using SPSS for Windows version 23.0 (IBM Corp., NY, USA). The associations with future RA were analysed in conditional logistic regression models with calculated odds ratios (ORs) and 95% confidence intervals (CIs). All *p* values are two-sided and *p* < 0.05 was considered statistically significant. Cardiovascular risk factors were selected according to previous studies and scoring systems as risk for CVD, defined as risk if presence of the following are present: hypertension (systolic blood pressure (SBP) ≥ 140 mmHg and/or diastolic blood pressure (DBP) ≥ 90 mmHg, including hypertensive treatment), diabetes (self-reported in questionnaires), BMI ≥ 25.0, elevated ApoB/ApoA1 ratio (women > 0.7, men > 0.8, including lipid lowering therapy) and smoking habits (analysed as current, ex and ever smoker but presented as ever smoker). A risk factor profile was defined as the number of risk factors present (having no risk factor as reference, or having one, two, or three or more) modified from the study by Berry et al. [21]. Results from analyses of HDL-cholesterol and LDL-cholesterol were lacking for approximately two-thirds of the individuals, whereas for the risk factors for CVD chosen to analyse further for associations with future development of RA, data on BMI, blood pressure, diabetes and smoking were available for 98.0–99.5% of the individuals and results of the ApoA1 and ApoB levels were available for 83% of the cases and 85% of the controls. Missing data for the ApoB/ApoA1 ratio was constructed using five imputations modelled by all data on CV risk factors, sex and age using the SPSS 23.0 program (IBM Corp.). Results from the original data are presented.

## Results

Smoking, either as current, ex or ever, were all significantly more prevalent among pre-symptomatic individuals compared with controls (OR (95% CI) = 2.74 (2.15–3.51), 1.52 (1.19–1.95) and 2.01 (1.64–2.47), respectively) (Table 1). This variable was therefore dichotomized into never or ever smokers in subsequent analyses.

**Table 1:** Results from conditional logistic regression analyses of pre-symptomatic individuals compared with controls presented for all individuals and stratified for women and men, respectively

	Pre-symptomatic individuals (*n* = 547)	Controls (*n* = 1641)	OR (95% CI)	Pre-symptomatic women (*n* = 372)	Control women (*n* = 1116)	OR (95% CI)	Pre-symptomatic men (*n* = 175)	Control men (*n* = 525)	OR (95% CI)
Smoking ever^a^	366/540 (67.8)	823/1609 (51.1)	2.01 (1.64–2.47)	241/366 (65.8)	548/1096 (50.0)	1.92 (1.50–2.47)	125/174 (71.8)	275/513 (53.6)	2.23 (1.53–3.27)
Current smoker^a^	197/538 (36.6)	326/1603 (20.3)	2.74 (2.15–3.51)	133/365 (36.4)	245/1094 (22.4)	2.01 (1.54–2.61)	64/173 (37.0)	81/509 (15.9)	3.11 (2.09–4.64)
Ex smoker^a^	167/538 (31.0)	491/1603 (30.6)	1.52 (1.19–1.95)	107/365 (29.3)	301/1094 (27.5)	1.56 (1.16–2.10)	60/173 (34.7)	190/509 (37.3)	1.49 (0.97–2.30)
Oral tobacco (snuff)	102/494 (20.6)	278/1491 (18.6)	1.22 (0.91–1.63)	22/329 (6.7)	77/998 (7.7)	0.93 (0.56–1.55)	80/165 (48.5)	201/493 (40.8)	1.40 (0.97–2.00)
BMI (kg/m^2^)^b^	25.7 (23.1–28.6) (*n* = 544)	25.3 (23.1–28.1) (*n* = 1634)	1.03 (1.00–1.05)	25.5 (22.8–28.6) (*n* = 369)	25.5 (22.8–28.6) (*n* = 1111)	1.03 (1.00–1.06)	26.0 (24.0–28.4) (*n* = 175)	26.0 (24.0–28.4) (*n* = 523)	1.02 (0.98–1.07)
Waist^c^ (cm)	93.0 (82.0–102.0) (*n* = 103)	89.0 (81.0–97.0) (*n* = 307)	1.02 (1.00–1.04)	85.5 (78.8–100.0) (*n* = 70)	85.0 (78.0–93.0) (*n* = 204)	1.02 (1.00–1.04)	98.0 (93.5–107.0) (*n* = 33)	96.0 (90.0–102.0) (*n* = 103)	1.03 (0.99–1.07)
Low education level^b,d^	452/540 (83.7)	1273/1613 (78.9)	1.41 (1.07–1.84)	294/366 (80.3)	854/1101 (77.6)	1.21 (0.88–1.65)	158/174 (90.8)	419/512 (81.8)	2.15 (1.23–3.78)
Hypertensive treatment	76/451 (16.9)	242/1333 (18.2)	0.84 (0.61–1.15)	47/306 (15.4)	168/899 (18.7)	0.70 (0.47–1.04)	29/145 (20.0)	74/434 (17.1)	1.20 (0.70–2.07)
Lipid lowering treatment	14/390 (3.6)	47/1164 (4.0)	0.73 (0.36–1.47)	6/266 (2.3)	24/773 (3.1)	0.46 (0.15–1.42)	8/124 (6.5)	23/391 (5.9)	1.06 (0.42–2.68)

Data presented as *n* (%) or median (Q1–Q3)

Odds ratio (*OR*) with 95% confidence interval (*CI*) in conditional logistic regression

*BMI* body mass index

^a^Reference group being never smoker

^b^OR per unit of increase

^c^Waist as continuous variable

^d^Low education level = no academic education/university

The pre-symptomatic individuals had a significantly higher BMI (analysed as a continuous variable) compared with controls in conditional logistic regression models (OR = 1.03 (1.00–1.05)) (Table 1). When stratified for sex, BMI remained significantly higher in the pre-symptomatic women compared with matched controls (OR = 1.03 (1.00–1.06)) (Table 1). The same pattern was observed for waist circumference, with significantly greater circumference for cases compared with controls, remaining significant for women when stratified for sex (Table 1).

A low educational level (no academic/university qualification) was associated with an increased risk for future RA (OR = 1.41 (1.07–1.84)), which was restricted to men (OR = 2.15 (1.23–3.78)) (Table 1).

### Lipid levels

Higher levels of ApoA1 were associated with a lower risk for future RA (OR = 0.50 (0.31–0.80)), and this was restricted to women when stratifying for sex (OR = 0.49 (0.28–0.85)) (Table 2). The ApoB/ApoA1 ratio (analysed as a continuous variable) was associated with risk for future RA with a higher ratio among pre-symptomatic women compared with controls (OR = 2.25 (1.25–4.06)) (Table 2). In conditional logistic regression analyses there were no significant differences in the levels of total cholesterol, HDL-cholesterol, LDL-cholesterol or triglycerides in cases compared with controls (Table 2). There were also no differences in these factors after stratification for sex (Table 2).

**Table 2:** Plasma glucose, blood pressure, total cholesterol, HDL-cholesterol, LDL-cholesterol, ApoA1 and ApoB in pre-symptomatic individuals and controls

	Pre-symptomatic individuals (*n* = 547)	Controls (*n* = 1641)	OR (95% CI)	Pre-symptomatic women (*n* = 372)	Control women (*n* = 1116)	OR (95% CI)	Pre-symptomatic men (*n* = 175)	Control men (*n* = 525)	OR (95% CI)
Plasma glucose, 0 hours (mmol/L)^a^	5.40 (5.05–5.84) (*n* = 513)	5.40 (5.00–5.80) (*n* = 1543)	1.11 (1.00–1.22)	5.40 (5.00–5.80) (*n* = 349)	5.38 (5.00–5.80) (*n* = 1052)	1.05 (0.92–1.21)	5.50 (5.10–6.00) (*n* = 164)	5.50 (5.10–5.90) (*n* = 491)	1.18 (1.01–1.37)
Plasma glucose, 2 hours (mmol/L)^a^	6.60 (5.70–7.30) (*n* = 470)	6.70 (5.90–7.60) (*n* = 1473)	0.94 (0.88–1.01)	6.67 (5.80–7.4) (*n* = 327)	6.80 (6.00–7.70) (*n* = 1005)	0.92 (0.85–1.00)	6.38 (5.40–7.10) (*n* = 143)	6.40 (5.40–7.40) (*n* = 468)	0.98 (0.87–1.10)
Systolic blood pressure (mmHg)^a^	128 (118–140) (*n* = 542)	129 (118–140) (*n* = 1637)	1.00 (0.99–1.01)	128 (115–140) (*n* = 368)	127 (118–140) (*n* = 1113)	1.00 (0.99–1.01)	128 (120–139) (*n* = 174)	130 (120–140) (*n* = 524)	1.00 (0.99–1.01)
Diastolic blood pressure (mmHg)^a^	80 (70–88) (*n* = 542)	80 (72–86) (*n* = 1635)	1.00 (0.99–1.01)	79 (70–87) (*n* = 368)	80 (70–85) (*n* = 1112)	1.00 (0.98–1.01)	80 (75–90) (*n* = 174)	80 (75–90) (*n* = 523)	1.00 (0.99–1.02)
Total cholesterol (mmol/L)^a^	5.74 (5.01–6.49) (*n* = 535)	5.70 (4.93–6.50) (*n* = 1619)	1.04 (0.96–1.14)	5.74 (5.01–6.57) (*n* = 361)	5.71 (4.94–6.57) (*n* = 1103)	1.04 (0.93–1.16)	5.76 (5.03–6.46) (*n* = 174)	5.70 (4.90–6.48) (*n* = 516)	1.06 (0.91–1.23)
Triglycerides (mmol/L)^a^	1.20 (0.89–1.60) (*n* = 453)	1.18 (0.84–1.70) (*n* = 1390)	0.95 (0.82–1.10)	1.16 (0.83–1.56) (*n* = 312)	1.14 (0.80–1.60) (*n* = 960)	0.95 (0.79–1.15)	1.29 (0.96–1.70) (*n* = 141)	1.36 (0.95–1.87) (*n* = 430)	0.94 (0.75–1.18)
HDL-cholesterol (mmol/L)^a^	1.26 (1.04–1.51) (*n* = 152)	1.32 (1.08–1.60) (*n* = 517)	0.81 (0.46–1.46)	1.30 (1.10–1.54) (*n* = 100)	1.40 (1.19–1.66) (*n* = 341)	0.65 (0.32–1.34)	1.19 (0.92–1.40) (*n* = 52)	1.14 (0.96–1.39) (*n* = 176)	1.60 (0.49–5.22)
LDL-cholesterol (mmol/L)^a^	4.13 (3.15–4.76) (*n* = 143)	4.07 (3.19–4.79) (*n* = 474)	1.12 (0.90–1.41)	4.08 (3.13–4.61) (*n* = 96)	4.02 (3.20–4.79) (*n* = 320)	1.06 (0.80–1.40)	4.28 (3.60–5.06) (*n* = 47)	4.14 (3.17–4.84) (*n* = 154)	1.25 (0.85–1.83)
ApoA1 (g/L)^a^	1.39 (1.23–1.57) (*n* = 453)	1.42 (1.28–1.59) (*n* = 1389)	0.50 (0.31–0.80)	1.46 (1.28–1.60) (*n* = 304)	1.47 (1.33–1.63) (*n* = 943)	0.49 (0.28–0.85)	1.31 (1.15–1.46) (*n* = 149)	1.32 (1.20–1.47) (*n* = 446)	0.53 (0.22–1.30)
ApoB (g/L)^a^	1.05 (0.86–1.24) (*n* = 453)	1.02 (0.86–1.21) (*n* = 1390)	1.50 (0.96–2.36)	1.02 (0.84–1.22) (*n* = 304)	1.00 (0.84–1.17) (*n* = 944)	1.66 (0.95–2.91)	1.11 (0.93–1.28) (*n* = 149)	1.09 (0.91–1.27) (*n* = 446)	1.24 (0.58–2.67)
Ratio ApoB/ApoA1	0.75 (0.60–0.93) (*n* = 453)	0.71 (0.58–0.88) (*n* = 1389)	2.05 (1.28–3.29)	0.70 (0.57–0.87) (*n* = 304)	0.67 (0.56–0.82) (*n* = 943)	2.25 (1.25–4.06)	0.86 (0.69–1.03) (*n* = 149)	0.82 (0.67–1.00) (*n* = 446)	1.74 (0.79–3.83)

Data presented as median (Q1–Q3)

Odds ratio (*OR*) and 95% confidence interval (*CI*) for future development of rheumatoid arthritis calculated with conditional logistic regression analyses for continuous variables in univariable analyses

*Apo* apolipoprotein, *HDL* high-density lipoprotein, *LDL* low-density lipoprotein

^a^OR per unit of increase

### Plasma glucose and blood pressure

The cases had significantly higher plasma glucose levels at baseline, and after stratification for sex the association with higher risk for future RA was restricted to men (OR = 1.11 (1.00–1.22) and 1.18 (1.01–1.37), respectively) (Table 2). Plasma glucose levels at 2 hours showed no differences in pre-symptomatic individuals compared with controls, and neither did the systolic or diastolic blood pressure (Table 2).

### Risk factors for CVD

Results from univariable conditional logistic regression analyses for selected risk factors for CVD (analysed as dichotomous variables), including elevated ApoB/ApoA1 ratio (men ≥0.8 compared with <0.8, women ≥0.7 compared with <0.7), ever smoking (vs never smoking), BMI ≥ 25 (vs BMI < 25.0), diabetes (vs no diabetes) and hypertension (systolic blood pressure (SBP) ≥ 140 mmHg and/or diastolic blood pressure DBP ≥ 90 mmHg including treatment for hypertension compared with SBP < 140 mmHg and DBP < 90 mmHg and no ongoing hypertensive treatment), are presented in Table 3. All factors, except hypertension, were significantly more frequent among the pre-symptomatic individuals compared with controls (Table 3). In the multivariable model, the factors remaining significantly associated with increased risk for future RA were smoking and BMI ≥ 25.0 (OR = 1.86 (1.48–2.35) and OR = 1.28 (1.01–1.62), respectively) (Table 3), which also remained significant after adjustment for educational level (data not shown).

**Table 3:** Odds ratio (95%) for association of risk factors for cardiovascular disease with future RA from univariable and multivariable conditional logistic regression models

	Elevated ApoB/ApoA1^a^	Smoking, ever^b^	BMI ≥ 25.0^c^ (kg/m^2^)	Diabetes	Hypertension^d^
Cases	241 (52.9)	366 (67.8)	322 (59.9)	17 (3.1)	78 (14.3)
Controls	667 (47.0)	832 (51.3)	850 (52.7)	27 (1.6)	268 (16.3)
Univariable	1.25 (1.01–1.56)^*^	2.00 (1.63–2.47)^***^	1.33 (1.09–1.63)^**^	1.98 (1.06–3.68)^*^	0.89 (0.72–1.11)
Multivariable	1.19 (0.95–1.50)	1.86 (1.48–2.35)^***^	1.28 (1.01–1.62)^*^	1.75 (0.86–3.58)	0.87 (0.67–1.11)
Univariable, women	1.41 (1.09–1.84)^*^	1.92 (1.50–2.47)^***^	1.34 (1.06–1.71)^*^	1.11 (0.46–2.67)	1.01 (0.77–1.32)
Multivariable, women	1.36 (1.03–1.80)^*^	1.82 (1.37–2.41)^***^	1.16 (0.87–1.54)	1.11 (0.41–2.99)	0.99 (0.72–1.34)
Univariable, men	0.99 (0.68–1.43)	2.23 (1.53–3.27)^***^	1.31 (0.91–1.90)	4.73 (1.71–13.08)^**^	0.76 (0.52–1.09)
multivariable, men	0.94 (0.63–1.40)	1.92 (1.26–2.92)^**^	1.56 (1.01–2.39)^*^	3.62 (1.13–11.64)^*^	0.67 (0.43–1.03)
Univariable, age ≤ 50.19 years	1.51 (1.11–2.06)^**^	2.20 (1.62–2.98)^***^	1.45 (1.09–1.93)^*^	2.09 (0.56–7.84)	0.88 (0.62–1.26)
Multivariable, age ≤ 50.19 years	1.39 (1.00–1.93)^*^	2.11 (1.51–2.95)^***^	1.45 (1.04–2.02)^*^	1.34 (0.28–6.27)	0.79 (0.53–1.19)
Univariable, age > 50.19 years	1.18 (0.86–1.62)	1.93 (1.43–2.60)^***^	1.21 (0.91–1.62)	2.10 (1.02–4.36)^*^	0.91 (0.68–1.20)
Multivariable, age > 50.19 years	0.90 (0.65–1.26)	1.74 (1.24–2.45)^**^	1.09 (0.77–1.55)	2.07 (0.90–4.81)	0.90 (0.65–1.26)

Data shown for all individuals overall, stratified for sex and age at 50.19 years

Data presented as *n* (%) or odds ratio (95% confidence interval)

*Apo* apolipoprotein, *BMI* body mass index, *RA* rheumatoid arthritis

^a^Males > 0.8, females > 0.7 including lipid lowering therapy

^b^Smoking ever compared with never smoking

^c^BMI ≥ 25.0 compared with BMI < 25.0

^d^Systolic blood pressure (SBP) ≥ 140 mmHg and/or diastolic blood pressure (DBP) ≥ 90 mmHg including treatment for hypertension compared with SBP < 140 mmHg and DBP < 90 mmHg and no ongoing hypertensive treatment

^*^ *p* ≤ 0.05,^**^ *p* ≤ 0.01, ^***^ *p* ≤ 0.001, dependent variable case/control

Stratification based on sex showed that in women the factors significantly associated with increased risk for future RA in the univariable models were smoking (OR = 1.92 (1.50–2.47)), elevated ApoB/ApoA1 ratio (OR = 1.41 (1.09–1.84)) and BMI ≥ 25 (OR = 1.34 (1.06–1.71)). In the multivariable model, smoking and elevated ApoB/ApoA1 ratio remained significantly associated with increased risk for subsequent RA (OR = 1.82 (1.37–2.41) and OR = 1.36 (1.03–1.80), respectively) in women (Table 3), which was unchanged after adjustment for educational level (data not shown). For pre-symptomatic men the significant factors in univariable models were smoking (OR = 2.23 (1.53–3.27)) and diabetes (OR = 4.73 (1.71–13.08)) (Table 3). In the multivariable model the same factors (i.e. smoking and diabetes) were significantly associated with increased risk for future RA in pre-symptomatic men (OR = 1.92 (1.26–2.92) and 3.62 (1.13–11.64), respectively), and additionally BMI ≥ 25.0 (OR = 1.55 (1.01–2.39)) (Table 3), which remained significant after adjustment for education level; furthermore, analyses showed that low educational level was associated with increased risk for subsequent RA in men (OR = 1.82 (1.02–3.26)) (data not shown).

Inclusion of age as a continuous variable in the conditional logistic regression analyses did not alter the results of the CVD risk factors associated with increased risk for future RA (data not shown). However, age was shown to be a significant factor associated with later RA development. Therefore, analyses were performed where age was stratified according to the median age at 50.19 years to receive two groups similar in size. These analyses showed that most of the risk factors for CVD were only significantly associated with increased risk for subsequent RA among younger pre-symptomatic individuals (Table 3). BMI ≥ 25.0, elevated ApoB/ApoA1 ratio and smoking were all significantly associated with increased risk for development of RA in multivariable conditional logistic regression models in younger individuals (OR = 1.45 (1.04–2.02), OR = 1.39 (1.00–1.93) and OR = 2.11 (1.51–2.95), respectively) (Table 3). In older individuals (>50.19 years), only smoking remained significant in the multivariable model (OR = 1.74 (1.24–2.45)) (Table 3).In older individuals (>50.19 years), only smoking remained significant in the multivariable model (OR = 1.74 (1.24–2.45)) (Table 3).

A variable with combinations of the five selected CVD risk factors was computed. The differences in frequencies between cases and controls of the combinations of no (reference), one, two or ≥ 3 of these risk factors for CVD were significant (χ^2^ = 24.26, *p* = 2 × 10^–5^). Of the pre-symptomatic individuals, 41.5% had three or more risk factors compared with 30.4% of the controls (χ^2^ = 19.17, *p* = 1 × 10^–5^). Corresponding numbers for male cases and controls were 47.0% and 39.1% (χ^2^ = 7.80, *p* = 0.005) and for female cases and controls were 38.7% and 26.3% (χ^2^ = 13.05, *p* = 3 × 10^–4^), respectively. Having one, two or ≥3 CVD risk factors were all significantly associated with future RA, with the highest OR for three or more risk factors (OR = 2.81 (1.78–4.44)) and significant both for women and men (OR = 2.63 (1.59–4.36) and 5.09 (1.48–17.54), respectively) (Table 4).

**Table 4:** Multivariable conditional logistic regression analyses for subsequent RA stratified for number of risk factors for cardiovascular disease^a^ present, stratified for median age (50.19 years) and sex

	No risk factor	One risk factor	Two risk factors	≥3 risk factors
All	Reference	1.73 (1.10-2.73)^*^	1.85 (1.17-2.94)^**^	2.81 (1.78-4.44)^***^
Age ≤ 50.19 years	Reference	1.80 (1.04-3.14)^*^	2.02 (1.14-3.57)^*^	4.00 (2.25-7.13)^***^
Age > 50.19 years	Reference	1.58 (0.65-3.87)	1.63 (0.69-3.83)	2.08 (0.90-4.85)
Women	Reference	1.47 (0.89-2.42)	1.55 (0.94-2.58)	2.63 (1.59-4.36)^***^
Men	Reference	3.92 (1.10-13.91)^*^	4.12 (1.18-14.36)^*^	5.09 (1.48-17.54)^*^

Data presented as odds ratio (95% confidence interval)

*Apo* apolipoprotein, *BMI* body mass index, *RA* rheumatoid arthritis

^a^Risk factors for cardiovascular disease: elevated ApoB/ApoA1 ratio (males > 0.8, females > 0.7 including lipid lowering therapy), smoking ever, diabetes (self-reported in questionnaires), hypertension (systolic blood pressure ≥ 140 mmHg and/or diastolic blood pressure ≥ 90 mmHg including treatment for hypertension), BMI ≥ 25

^*^ *p* ≤ 0.05, ^**^ *p* ≤ 0.01, ^***^ *p* ≤ 0.001, dependent variable case/control. Pre-symptomatic individuals compared with controls (reference being no risk factor present), stratified for age ≤ 50.19 years or > 50.19 years and women/men

Also, the younger pre-symptomatic individuals had significantly higher ORs for combinations of CVD risk factors (OR = 1.80 (1.04–3.14), 2.02 (1.14–3.57) and 4.00 (2.25–7.13) for one, two or ≥3 of the factors, respectively, with no risk factor as the reference group), whereas none of the combinations of CVD risk factors were significant among the older individuals (Table 4).

### Sensitivity analyses

Sensitivity analyses were performed on pre-symptomatic individuals who had experienced symptoms ≤1 year within recruitment into the VIP or MONICA study. This showed that there were no significant differences between them and the rest of the cases regarding age, sex, BMI, ApoB/ApoA1 ratio and systolic or diastolic blood pressure. In the univariable analyses, the association of elevated ApoB/ApoA1 ratio was weakened by exclusion of cases with pre-symptomatic duration ≤ 1 year. However, when stratified for sex the associations were unchanged.

In the multivariable analysis, the association for BMI ≥ 25 for later RA development was altered (OR (95% CI) = 1.24 (0.98–1.58)) and when stratified for sex the significance among male cases was lost (OR (95% CI) = 1.43 (0.92–2.22), *p* = 0.11). Sensitivity analysis after imputation of data for ApoB/ApoA1 did not show any differences for the cardiovascular risk factors, nor for age or sex distributions.

## Discussion

In this study we have shown that individuals who subsequently develop RA have increased levels and frequencies of CVD risk factors years before onset of RA. Some of the CVD risk factors prevalent in the pre-symptomatic individuals (e.g. smoking and elevated BMI) have previously been identified as potential risk factors for RA [12, 15, 16].

We have shown that smoking is related to an increased risk of future RA regardless of sex or age with a two-fold increased risk. The other risk factors showed differences when stratified for sex or age. In women the significant risk factor other than smoking was elevated ApoB/ApoA1 ratio, whilst among male cases BMI ≥ 25.0 and diabetes were associated with an increased risk. Diabetes had a four-fold risk, which was the highest of all risk factors studied. In the cohort study by Lahiri et al. [17], being a current smoker was associated with an increased risk of future RA or IP in men, whilst diabetes was associated with an increased risk of RA in women. In the general population, smoking and diabetes have a slightly greater effect in women compared with men for risk of CVD [22], even though male sex is reported to be a risk for developing type 2 diabetes [23].

The relationship between obesity and RA has proved contradictory; a recent meta-analysis of 11 studies concluded that an increased BMI could contribute to higher risk for RA [15]. Recently, we published a study focusing on obesity as a possible risk factor for future RA development based on data from the same cohorts as this study, showing that obesity or abdominal obesity was associated with risk of future RA, mainly in men with an early disease onset [16]. In the present study, a higher BMI was associated with increased risk for future RA, even when adjusting for smoking, ApoB/ApoA1 ratio, diabetes, hypertension and educational level, although, when stratifying for sex, the association of BMI ≥ 25.0 for future RA was restricted to men. The association of being overweight or obese with the risk of RA could potentially be due to both conditions being linked with inflammation, and consequently with increased levels of inflammatory cytokines. It can be hypothesized that one reason why BMI ≥ 25.0 was not associated with a significant risk for future RA among women in our study was that among these pre-symptomatic individuals a relatively high proportion had yet to reach menopause, when overweight and obesity is known to increase. Additionally, in obese men accumulation of fatty tissue is mainly visceral, with more abundant pro-inflammatory cytokines, whereas in pre-menopausal women it is mainly subcutaneous [24].

Apolipoprotein A1 (ApoA1) is the major apolipoprotein in HDL, and higher levels are associated with a reduced risk for CVD; ApoB is the main apolipoprotein in LDL and therefore atherogenic. Several studies have shown that ApoB and the ratio of ApoB/ApoA1 are a better predictor for CVD and CV events than total cholesterol, LDL-cholesterol and cholesterol/HDL ratio [25–28]. In a study on patients with RA analysing possible predictors for CV events during 18 years, the ratio of ApoB/ApoA1 was predictive for CV events without association with inflammatory markers [29]. In this study we showed that the pre-symptomatic individuals had a significantly higher frequency of elevated ApoB/ApoA1 ratio compared with matched controls, and stratification for sex showed that the association with increased risk for future RA was restricted to women. Consistent with these results, a previous publication reported that blood donors who later developed RA had a more atherogenic lipid profile with higher total cholesterol, triglycerides and ApoB together with decreased HDL-cholesterol, with remaining levels after adjustment for CRP [13]. In a recent study, high serum levels of cholesterol in women were associated with future RA [30].

Hypertension was not more frequent among pre-symptomatic individuals compared with controls. One possible reason for this could be that pre-symptomatic individuals had not yet experienced symptoms of joint disease and, therefore, had no greater usage of NSAIDs, known to increase blood pressure, compared with the controls. The use of glucocorticoids in RA has also been associated with hypertension and could partly explain that there is no difference in the frequency of this condition in pre-symptomatic individuals compared with controls, because the pre-symptomatic individuals had not yet been prescribed any glucocorticoids. Furthermore, it has been suggested that a systemic inflammation with high CRP levels and elevated cytokines can lead to hypertension, as reviewed by Panoulas et al. [31]. Unfortunately, we had no information on CRP levels in the pre-symptomatic individuals but it could be speculated that levels had not reached those observed in newly diagnosed RA patients.

Because age, in addition to the risk factors, had an impact on the association of being pre-symptomatic, individuals were stratified for age. Both elevated ApoB/ApoA1 ratio and BMI ≥ 25.0 are associated with future development of RA in younger individuals. A recent meta-analysis studying whether the same impact of age and gender on CVD risk was seen in RA as in the general population, with the highest risk among men and older individuals, concluded that the risk of CVD is age dependent [32]. The highest relative risk for CVD was in the youngest RA patients [32]. Interestingly, the results in our study showed that the presence of most of the CVD risk factors was only significantly associated with increased risk among younger pre-symptomatic individuals. The same pattern was observed of having the combination of the CVD risk factors, which were only significantly more prevalent in younger pre-symptomatic individuals compared with matched controls.

One limitation of this study is that approximately 60% of the population participates in the studies at the Medical Biobank. Being male, non-EU country of birth, single living in rural areas, low educational level, low income, being hospitalized more than twice and prior CVD characterized the non-participants [33]. The lack of data from these individuals possibly has an impact on our analyses in several ways, both strengthening and weakening the associations. Additionally, a limitation is that individuals younger than 40 years when affected with RA would be lost from inclusion in our study. Furthermore, for some of the variables, such as information on HDL-cholesterol, LDL-cholesterol and waist circumference, data were lacking and therefore interpretation of these results should be made with caution.

The strengths of this study include the possibility to use data from a well-defined large population-based database incorporating individuals having previously participated by completing questionnaires and donating blood samples to the cohorts in the Medical Biobank prior to onset of symptoms of RA. The blood pressure and anthropometric measurements were assessed by a trained nurse in all individuals. Most of the analyses were undertaken with conditional logistic regression analyses because for each pre-symptomatic individual three controls were selected randomly from the same cohorts and matched for sex, date of birth and year of clinical examination, and rural or urban living area. The subsequent diagnosis of the cases was based on clinical examinations by a rheumatologist. The controls were not clinically examined for presence of joint disease, but until the end of 2014 (when the co-analysis of registers was performed) they were not diagnosed with RA.

We have analysed the associations of the CVD risk factors with the development of future RA, but it is tempting to speculate about the impact of the presence of the CVD risk factors years before onset of symptoms of RA and the known relationship between RA and cardiovascular co-morbidity and mortality [9].

## Conclusions

Our results show that several of the known CVD risk factors, of which several are per se related, are present in individuals prior to the onset of symptoms of RA. In pre-symptomatic women both smoking and an elevated ApoB/ApoA1 ratio were significantly more prevalent compared with matched controls, whereas in men smoking, BMI ≥ 25.0 and diabetes were significantly associated with increased risk of future development of RA. Also, the pre-symptomatic individuals had significantly higher frequencies of combinations of the CVD risk factors compared with matched controls, particularly the younger individuals. Together, these results urge a prompt analysis of CVD risk factors for proposed prevention in patients with early RA.

## Abbreviations

ACPA: Anti-citrullinated peptide antibodies
Apo: Apolipoprotein
BMI: Body mass index
CVD: Cardiovascular disease
HDL: High-density lipoprotein
LDL: Low-density lipoprotein
MI: Myocardial infarction
MONICA: Northern Sweden Multinational Monitoring of Trends and Determinants in Cardiovascular Disease
OR: Odds ratio
RA: Rheumatoid arthritis
RF: Rheumatoid factor
VIP: Västerbotten Intervention programme
